# The Dorsal Column Lesion Model of Spinal Cord Injury and Its Use in Deciphering the Neuron‐Intrinsic Injury Response

**DOI:** 10.1002/dneu.22601

**Published:** 2018-05-11

**Authors:** Callan L. Attwell, Mike van Zwieten, Joost Verhaagen, Matthew R. J. Mason

**Affiliations:** ^1^ Laboratory for Regeneration of Sensorimotor Systems Netherlands Institute for Neuroscience, an Institute of the Royal Netherlands Academy of Arts and Science, Meibergdreef 47 Amsterdam 1105BA The Netherlands; ^2^ Center for Neurogenomics and Cognitive Research, Vrije Universiteit Amsterdam, De Boelelaan 1085 Amsterdam 1081HV The Netherlands

**Keywords:** conditioning lesion, dorsal column lesion, dorsal root ganglia, regeneration‐associated gene program, spinal cord injury

## Abstract

The neuron‐intrinsic response to axonal injury differs markedly between neurons of the peripheral and central nervous system. Following a peripheral lesion, a robust axonal growth program is initiated, whereas neurons of the central nervous system do not mount an effective regenerative response. Increasing the neuron‐intrinsic regenerative response would therefore be one way to promote axonal regeneration in the injured central nervous system. The large‐diameter sensory neurons located in the dorsal root ganglia are pseudo‐unipolar neurons that project one axon branch into the spinal cord, and, via the dorsal column to the brain stem, and a peripheral process to the muscles and skin. Dorsal root ganglion neurons are ideally suited to study the neuron‐intrinsic injury response because they exhibit a successful growth response following peripheral axotomy, while they fail to do so after a lesion of the central branch in the dorsal column. The dorsal column injury model allows the neuron‐intrinsic regeneration response to be studied in the context of a spinal cord injury. Here we will discuss the advantages and disadvantages of this model. We describe the surgical methods used to implement a lesion of the ascending fibers, the anatomy of the sensory afferent pathways and anatomical, electrophysiological, and behavioral techniques to quantify regeneration and functional recovery. Subsequently we review the results of experimental interventions in the dorsal column lesion model, with an emphasis on the molecular mechanisms that govern the neuron‐intrinsic injury response and manipulations of these after central axotomy. Finally, we highlight a number of recent advances that will have an impact on the design of future studies in this spinal cord injury model, including the continued development of adeno‐associated viral vectors likely to improve the genetic manipulation of dorsal root ganglion neurons and the use of tissue clearing techniques enabling 3D reconstruction of regenerating axon tracts. © 2018 The Authors. Developmental Neurobiology Published by Wiley Periodicals, Inc. Develop Neurobiol 00: 000–000, 2018

## THE DORSAL COLUMN LESION MODEL

The neuronal response to axotomy differs greatly between the peripheral nervous system (PNS), where nerve injury results in the initiation of a robust growth program, and neurons of the central nervous system (CNS), where often little or no response occurs. This dichotomy is observed even within a single‐cell‐type, the large‐diameter sensory neurons of the dorsal root ganglia (DRG) which project both to the periphery and along the spinal cord to the brainstem. Injury to the peripheral branch induces the regeneration program, while injury to the central branch in the spinal cord generally does not, although injury to the dorsal root does produce a mild regenerative response.

**Figure 1:**
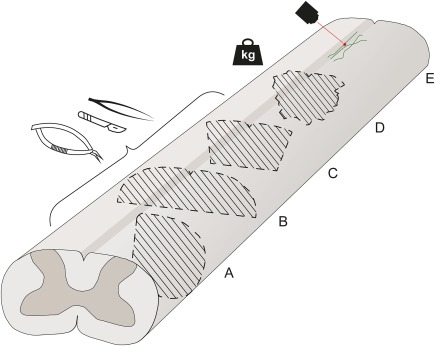
Commonly used rodent DC lesion models. A schematic diagram of the rat spinal cord and common DC lesion paradigms. Injured areas are depicted with striped lines, together with instruments commonly used to perform the lesion. (A–C) Transection injuries of the spinal cord, illustrating in (A) lateral hemisection of the spinal cord, (B) dorsal hemisection of the spinal cord, and (C) bilateral transection of the DC (microscissors and scalpel depicted, other instruments are also used as summarized in Table 1 and Supporting Information, Table 2). Besides transection, the DC lesion can be implemented using forceps creating a crush injury. (D) Contusion or compression injury by dropping or placing a weight on the spinal cord in a controlled manner. (E) Severing individual superficial DC axons using a laser. [Color figure can be viewed at http://wileyonlinelibrary.com]

**Figure 2:**
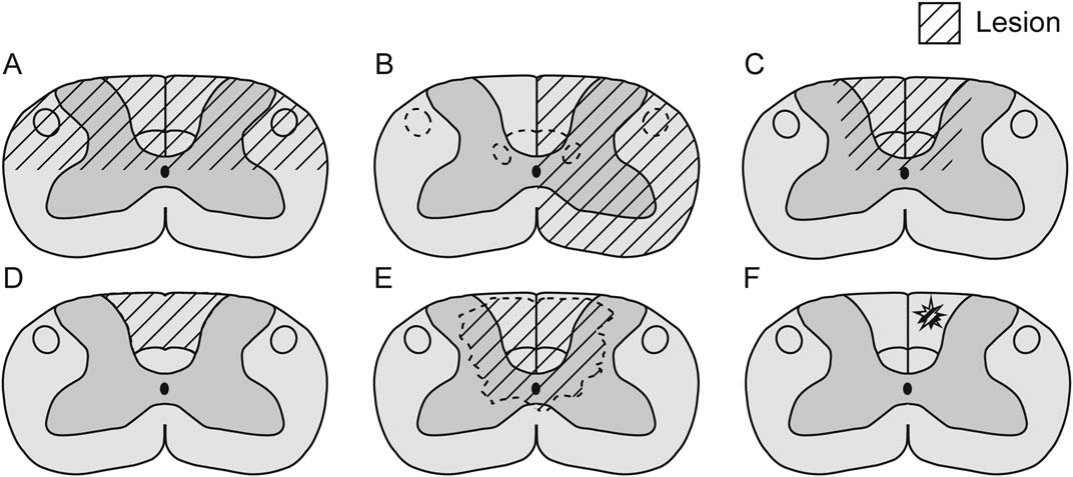
Detailed illustration of DC lesion models for SCI. Schematic drawings of transverse sections of adult rat cervical (C7) spinal cords (modified from Watson et al., 2008) depicted in the striped areas the injuries to the spinal cord with (A) dorsal hemisection of the spinal cord, (B) lateral hemisection of the spinal cord, (C) complete bilateral DC transection, (D) bilateral DC aspiration, (E) spinal cord contusion, and (F) single DC axon transection injuries.

Increasing the neuron‐intrinsic regenerative response is one approach to promote regeneration in the injured central nervous system. The dorsal column (DC) lesion model of spinal cord injury is ideally suited to the study of this topic and is the subject of this article. We will discuss its advantages and disadvantages as a spinal cord injury (SCI) model and in particular as a model for the study of neuron‐intrinsic regenerative capacity. We will cover the variety in injury techniques, which include transient compression or contusion, complete and partial transection, crush or specific transections of the dorsal columns (DCs) (Figs. 1 and 2), and the theoretical and observed pathways of axonal regeneration. We also review the results of experimental interventions, with emphasis on modulation of the neuron‐intrinsic regeneration response.

**Figure 3:**
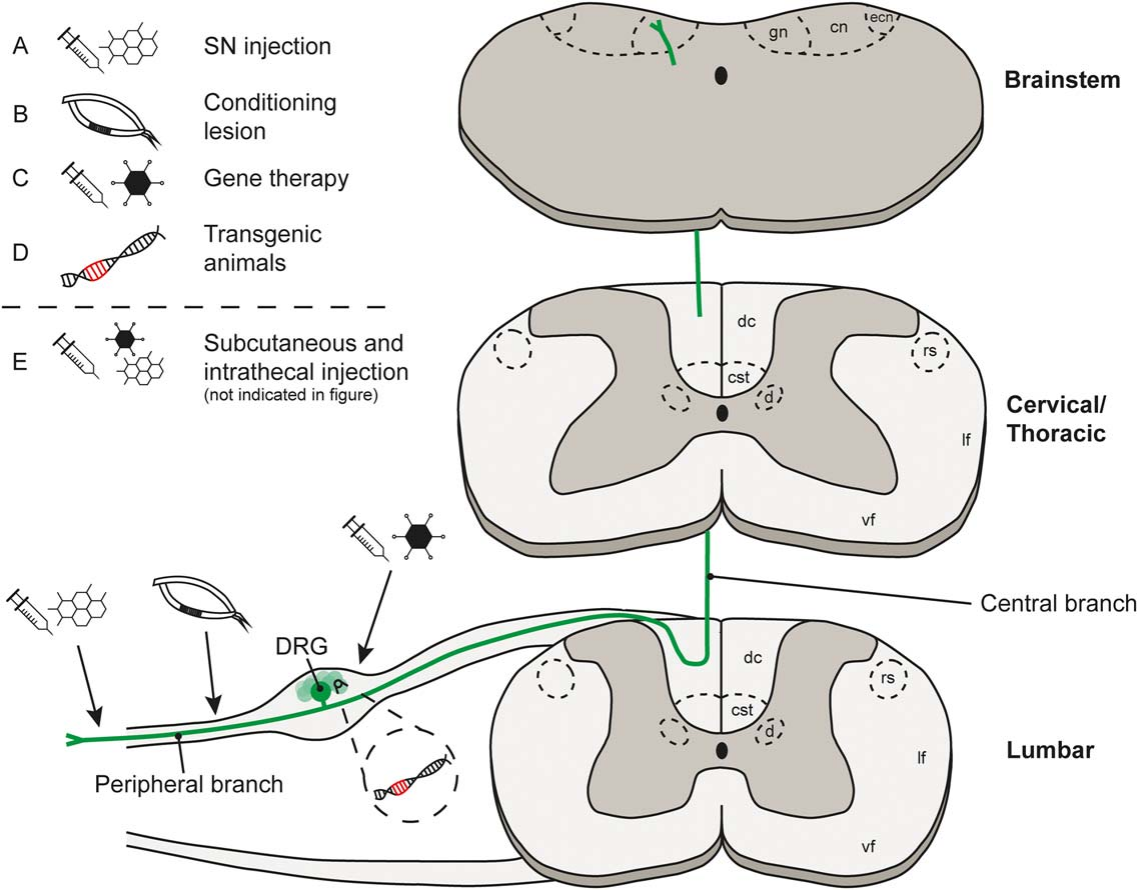
The anatomy of the dorsal root ganglia, the spinal cord and targets for neuron‐intrinsic experimental intervention strategies. Axons of DRG neurons bifurcate into two branches, one going into the periphery and the other going into the spinal cord. These axons relay information including heat, pain, and position from the body and project via the DC to the brainstem directly (the gracile nucleus, cuneate nucleus and the external cuneate nucleus) or indirectly via spinal neurons (not shown). Not shown: collaterals also innervate spinal cord grey matter in the segments around where they enter, and some collaterals descend caudally. Axon collaterals also innervate Clarke's nucleus in the thoracic cord. The arrows indicate targets for neuron‐intrinsic intervention to promote axonal growth and plasticity, with (A) introduction of pharmacological agents by injection into the SN, (B) CL of the SN, (C) viral vector delivery by injection into the DRG, (D) using transgenic animals, and (E) subcutaneous or intrathecal injection to deliver pharmacological agents (not illustrated). Key: gn = gracile nucleus, cn = cuneate nucleus, ecn = external cuneate nucleus, dc = dorsal column, cst = corticospinal tract, d = dorsal nucleus (Clarke's nucleus), rs = rubrospinal tract, lf = lateral funiculus, vf = ventral funiculus. [Color figure can be viewed at http://wileyonlinelibrary.com]

The DC contains axon branches from large‐diameter dorsal root ganglia neurons that ascend to the brainstem. DRG neurons are pseudo‐unipolar cells with an axon that divides to give rise to a peripheral branch, which innervates the periphery via the peripheral nerves, and a central branch that enters the spinal cord (SC) (Fig. 3). The peripheral axon branch receives somatic information such as discriminatory touch, vibration, proprioception, and tactile information (Sengul and Watson, 2014). The central branch of the DRG neurons carries this sensory information into the CNS, entering the spinal cord via the dorsal root entry zone (DREZ). Some of these axons ascend directly to the brainstem nuclei via the DC which in turn project to higher centers in the brain (Fig. 3). The central afferents also innervate second‐order neurons located in the grey matter of the spinal cord involved with unconscious proprioception, central pattern generator (CPG)‐associated stepping, and unconscious paw withdrawal to noxious stimuli (Basbaum et al., 2009; Sengul and Watson, 2014; Takakusaki, 2017).

The peripheral and the central branch of DRG neurons differ considerably in their response to injury, despite originating from the same cell body. The peripheral branches of DRG neurons show robust spontaneous regeneration after injury, whereas the central branches display relatively limited regeneration in response to a lesion of the dorsal roots and no regeneration upon transection of the DCs. The fact that central branches of DRG neurons show relatively limited spontaneous regeneration is likely due to a combination of neuron‐extrinsic and neuron‐intrinsic factors. After injury of the peripheral branch of the DRG neurons, a large number of regeneration associated genes (RAGs) are differentially regulated (Hoffman, 2010; van Kesteren et al., 2011; Smith and Skene, 1997; Stam et al., 2007). A number of prominent cytological changes also occur as part of the “cell body response” (Lieberman, 1971). Injury to central branches of DRG neurons elicits a weaker cell body response and limited RAG expression, depending on the location of the injury. Additionally, the failure of regeneration of central branches of DRG neurons after spinal cord injury is also partly caused by the unfavorable environment for growth at the lesion site, due to the presence of glial barriers (e.g., presence of reactive astrocytes; see Silver et al., 2015 for review) and inhibitory extracellular molecules (e.g., proteoglycans and myelin‐associated proteins; see Filbin, 2003 for review) that are present in scar tissue. The presence of macrophages around the lesion site, as part of the inflammatory response, also limits regeneration following CNS injury (Kigerl et al., 2010). Taken together, the hostile environment and failure to activate the RAG program after injury both contribute to the inability to elicit successful regeneration in ascending DC axons following SCI.

The different growth capacities of the peripheral and the central branches of DRG neurons make DRG neurons a unique target of study to acquire insight into the intrinsic molecular mechanisms underlying successful axon regeneration. Importantly, the intrinsic growth state of the DC axons, i.e. the central processes of DRG neurons, can be partially enhanced upon injury by a lesion of the peripheral branch, often referred to as a “conditioning lesion” (Hoffman, 2010; McQuarrie et al., 1977; Neumann and Woolf, 1999; Qiu et al., 2002; Richardson and Issa, 1984). A conditioning lesion (CL) of the peripheral nerve allows a limited degree of axon regeneration of ascending sensory afferents in the DC after a DC lesion. CLs have been implemented prior to a DC lesion, together with a transplantation of the peripheral nerve into the lesion site resulting in robust regeneration into the peripheral nerve graft (Oudega et al., 1994; Richardson and Issa, 1984).

Following lesioning and intervention, this model has the useful property that it is possible to verify the completeness of the lesion with regard to transganglionically labeled fibers by investigation of the brainstem nuclei to determine the presence or absence of spared fibers. This in principle removes the uncertainty of whether one is observing actual regeneration or sprouting of intact fibers beyond the lesion, something that is difficult in other models such as corticospinal tract (CST) lesions. The unique properties of the DRG neuron, in conjunction with a DC lesion and reliable tracing provide an excellent model to study the genetic and molecular factors underlying processes which regulate neuroregeneration.

## EXPERIMENTAL LESION MODELS FOR DORSAL COLUMN INJURY

There are considerable differences in the surgical approaches used to generate DC lesions in the literature. Transection, crush, and contusion lesions are all in use (illustrated in Fig. 1). There is also variation within each technique, such as the anatomical level, the depth of transection or crush, the type of instruments used, and force or weight applied in contusion models. Further differences arise from the use of bridging or grafting and closure of the lesion. While lesioning of the DC will result in loss of sensory function and, often, motor function due to damage caused to other spinal tracts including the descending motor axons of the CST which lies immediately beneath the DC, the severity of the functional deficit will depend on the extent of the lesion and the exact lesion technique. Additional factors that can affect the assessment of the regenerative response or functional recovery include the species, age, functional test, survival time, and type of histology and quantification methodology.

Here we have attempted to produce a comprehensive summary table of studies which focused on DC lesion surgical techniques or assessment of functional recovery (Table 1) and research where a DC lesion was used with an experimental intervention (Supporting Information, Table 2). The type of DC lesion, surgical instruments and methodology used, experimental intervention, quantification of axonal regeneration, axon retraction, plasticity and, where performed, assessment of functional recovery have been briefly summarized.

**Table 1:** Studies on Dorsal Column Lesion Methods and Functional Testing Following Dorsal Column Lesions

Paper	Type of Dorsal Column (DC) Lesion	Level	Species	Instrument	Surgical Detail	Weeks Survival After Lesion	Histology, Functional Tests	Result Summary	Last Author(s)^a^
Onifer et al., 2005	DC: Bilateral complete DC transection DF: Bilateral complete DF transection DLF: Bilateral complete dorsolateral funiculi transection DHx: C4 dorsal hemisection	C4	Rat	DC, DF: Custom‐made 0.12‐mm‐thick and 1.6‐mm‐wide diamond‐shaped piece of razor blade attached to the Vibraknife, a modified VIBRATOMER Series 1000 DLF: 0.8‐mm‐wide piece of razor blade/Vibraknife DHx: 0.8‐mm‐wide piece of razor blade/Vibraknife	DC: Laceration lesion to a depth of 1.1 mm DF: Laceration lesion to a depth of 1.5 mm DLF: “Left and right dorsolateral funiculi, cut a depth of 1.5 mm, and maximum 1 mm medially from the cord's lateral edge. DHx: Laceration lesion to a depth of 1.5 mm	DC, DF, DLF:1 DHx:4	Transganglionic axonal tracing with CTB DC: Sticker attention, electrophysiological assessment after behavioral tests (1 week after SCI) DF: Sticker attention, electrophysiological assessment after behavioural tests (1 week after SCI) DLF: Sticker attention, electrophysiological assessment after behavioral tests (1 week after SCI) DHx: (a) Sticker attention, electrophysiological assessment after behavioral tests (1 week after SCI), (b) weekly sticker attention and grid walking with electrophysiological assessment (4 weeks after SCI), (c) sticker attention and electrophysiological assessment (4 weeks after SCI)	An increase in reaction time to sticker attention in DC, DF, but not in DLF lesioned animals after 1 week. Returns to baseline after a week. DHx: Gridwalk increase footfall persisted at 4 weeks. Sticker attention delayed when measured only at 4 weeks. Somatosensory‐evoked potentials were abolished in complete DC and DF lesions, but not DLF lesions.	Magnuson
Fagoe et al., 2016	Bilateral transection	C4, T7	Rat	30G needle, 27G needle, microscissors	To minimize compression damage of the spinal cord a 30G needle was inserted at 1 mm (at C4) or 0.6 mm (at T7) lateral to the midline on either side to a depth of 1.6 mm (at C4) or 1.4 mm (at T7). The resulting hole was then enlarged by inserting a 27G needle to the same depth. Finally the tips of a pair of micro‐scissors were inserted in the same holes to the same depth and then closed.	8	Transganglionic axonal tracing with CTB, gracile nucleus assessed for spared axons. Adhesive tape removal test, horizontal ladder, rope crossing test, CatWalk gait analysis and the inclined rolling ladder. Hindlimbs assessed only.	Deficits on tape removal and rope tests were minor and short‐lived. C4 lesions were more severe than T7. Inclined rolling ladder (C4) gave deficits for 6 weeks, Horizontal ladder (C4) deficits small but significant over time‐course. Catwalk parameters affected, mainly by C4 lesion. Generally, function recovered to sham levels within 2–6 weeks.	Mason
Kanagal and Muir, 2008	Bilateral transection	C2, T7–8	Rat	25G hypodermic needle	Using a modified sterile 25G hypodermic needle, lesions were made bilaterally to either the cervical (C2) or mid‐thoracic (T7–8) dorsal funiculus (DCs & dorsal corticospinal tract)	2, 6	Overground locomotion and horizontal ladder	C2 DC lesion resulted in persistent errors in both functional tests. In contrast, T7–8 DC lesion did not affect over ground locomotion and only caused minor errors in horizontal ladder at 2w postinjury, with recovery to presurgical levels 6 w after injury.	Muir
Kanagal and Muir, 2007	Bilateral transection	C2, T7–8	Rat	25G hypodermic needle	Using a modified sterile 25G hypodermic needle, lesions were made bilaterally at cervical (C2) to either DCs alone or DCs and CST. Autologous fat graft placed over laminectomy site to prevent fibrous adhesions to the spinal cord and dura.	8	Horizontal ladder, skilled reaching, overground locomotion, ground reaction force (GRF).	DC+ CST lesions resulted in more serious deficits, detectable in all tests up to 8 weeks. DC lesion alone resulted in detectable deficits up to 4 weeks on horizontal ladder. Deficits were detectable in GFR and limb timing at 8 weeks in both groups but no difference between lesions suggesting these tests were sensitive to ascending sensory input alone.	Muir
Hill et al., 2009	Bilateral transection	T9	Mouse	Louisville Injury Systems Apparatus (LISA‐Vibraknife)	Four dorsal hemisection injuries with lesion depths of 0.5, 0.8, 1.1, and 1.4 mm, as well as normal, sham, and transection controls. Spinal column stabilized, and lesioned with LISA‐Vibraknife	6	BMS, footprint analysis, beam walk, toe spread reflex, Hargreaves and transcranial magnetic motor‐evoked potential (tcMMEP)	Performance generally deteriorated more with higher depth of lesion. All tests showed deficits for the full 6 weeks in the deepest lesions and many even in the shallow lesions. Hindpaw response times were reduced on Hargreaves’ test.	Shields
Ballermann et al., 2001	Unilateral transection	C1	Rat	No. 11 razor blade	A sagittal incision was made in the nape of the neck, and the C1 and C2 vertebrae were exposed through blunt dissection. The medial part of the dorsal arch of C1 was removed with a drill, and the DC was cut using a sharp No. 11 razor blade. The transactions were made on the ipsilateral side to the preferred paw for reaching	1	Reaching task, force measurement, haptic discrimination, vertical paw placing, adhesive dot removal	Normal performance levels on all tests with the exception of deficits in haptic discrimination when feeling for a food/non‐food item.	Whishaw
McKenna and Whishaw, 1999	Unilateral transection	C2	Rat	No. 11 razor blade	A sagittal incision was made at the nape of the neck, blunt dissection revealed the dorsal surface of the first and second cervical vertebrae. The mediocaudal part of the C1 vertebra was removed with rongeurs, so that the DC tract on one side of the spinal cord was made visible and could be incised with a sharp No. 11 scalpel blade.	3	Reaching task and rotary limb movement analysis	Reaching success completely recovered within a few days of DC lesion. Compensation was achieved with whole‐body and alternate limb movements (which were irreversibly impaired).	Whishaw

a Papers arranged alphabetically by final author, most recent papers first.

### Transection Injury Models

Transection of the ascending sensory afferents in the DC can be performed by selectively transecting only the ascending afferents, or by more gross transections such as a dorsal or lateral hemisection of the cord that cause damage to other areas of the spinal cord. Transection lesions are ideal for studying anatomical regeneration of the DC, due to the ability to definitively transect the axons of interest with very little chance of sparing fibers, and relatively straightforward techniques to check for regeneration and sparing with tracers. The most relevant transection lesion for the study of regeneration of ascending afferents from the DRG is the selective DC transection, although this will of course result in partial or complete transection of the CST. The selective DC transection can be executed by bilaterally lesioning the dorsal funiculus of the spinal cord (Fig. 2A–C). Alternatively, a dorsal hemisection may be performed which lesions the DC and will also lesion both rubrospinal tracts (RSTs) and CSTs. A lateral hemisection of the spinal cord will unilaterally lesion the DC and all other spinal cord tracts on the sectioned side. Lesioning the DC by selective transection results in targeted interruption of the tract without complete interruption of the spinal cord, which minimizes the physical damage from the injury to other spinal cord tracts (Steward et al., 2003). Transection of the DC and hemisection of the spinal cord has been performed in many ways, as summarized in Table 1 and Supporting Information, Table 2. Following spinal column exposure, laminectomy and removal of the dura, transection techniques can be carried out at different levels of the spinal cord using different surgical tools, although most commonly with microscissors, and lesions can be either bilateral or unilateral.

Transection paradigms include, for example, dorsal hemisection at the lower thoracic level using a microknife (Tedeschi et al., 2016) or at the cervical level with a 3.8‐mm‐wide piece of razor blade (Onifer et al., 2005), lateral hemisection at the T11–12 level using microscissors (Tang et al., 2015) and DC transection at the high cervical level with a Scouten wire‐knife (Hollis et al., 2015b). In demonstrating the CL effect in a DC lesion, a bilateral complete DC transection up until the central canal was used (Neumann and Woolf, 1999), also severing the descending CST in rats. In our experiments, we first make holes in the cord either side of the midline with a 30 G needle, enlarge the holes with a 27 G needle and then insert the microscissor points into the holes and close them, bilaterally transecting the DCs. This has the aim of minimizing contusion damage to the cord while inserting the microscissors (Fagoe et al., 2016). Moon et al. (2006) directly performed a DC transection without laminectomy with microscissors at the naturally occurring gap between T10 and T11 to a depth of 1 mm. Additionally, the DC can also be cut incompletely, for example, superficially cutting individual DC axons (Fig. 2F) using microscissors (Ertürk et al., 2007; He et al., 2016) or two‐photon laser cutting (Fig. 1E; Ylera et al., 2009).

Transection lesions typically result in a gap at the lesion site. A common approach is to fill the lesion site with a cell graft to provide an extracellular matrix for physical support of the injured tract, and depending on the cell type, neurotrophic support. As summarized in Supporting Information, Table 2, various cell types have been used, including glial‐restricted precursors (GRPs; Bonner et al., 2011; Haas et al., 2012), bone marrow stromal cell (BMSCs; Hollis et al., 2015b), peripheral nerve implants when combined with a CL (Richardson and Issa, 1984), and olfactory ensheathing cells (OECs; Toft et al., 2007).

Although specific transection of the DC and dorsal and lateral hemisection injuries are rarely seen in human SCIs, these lesions allow researchers to answer questions regarding sprouting, die back and remodeling on an axonal level (Talac et al., 2004; see Brösamle and Huber, 2007 for review).

### Crush Injury Models

Another paradigm that is often used to lesion the spinal cord is crush injuries. These also resemble a more clinically relevant injury when compared to transection models. Usually crush injuries are performed by making holes either side of the DC with a very sharp instrument before a pair of forceps are inserted and closed for a certain amount of time to crush the DC while keeping the structural organization of the rest of the cord largely intact. The advantage that this has over transection lesions is that crushed spinal cord tissue provides a substrate for regenerating axons to traverse. This lesion model may be less susceptible to the formation of large cerebrospinal fluid‐filled cysts observed after transection lesion in rats (e.g., see Neumann and Woolf, 1999). However, due to the higher probability of sparing of fibers following a crush injury as compared to a transection injury, it is of great importance to use tracing to determine completeness of the lesion and exclude the animals with spared fibers from quantification.

As with transection lesions, no standardized procedure for crush lesions exists, and forceps may be inserted at variable width, depth, duration, and level of the spinal cord. Bradbury et al. (1999) inserted a pair of fine forceps to a depth of 2 mm bilaterally into the spinal cord which was held tightly for 10 s before raising them back out of the spinal cord. The reader will note the many surgical approaches for crush injuries listed in Supporting Information, Table 2, for example, Puttagunta et al. chose to make 2‐s‐long crush injuries of the DC with no. 5 forceps to a depth of 2 mm. Gaudet et al. (2016) selected a more specific and severe paradigm where they injured axons to a depth of 0.6 mm and held a pair of forceps tightly together for 10 s and repeated this three times (Puttagunta et al., 2014).

Crush lesions have also been carried out in conjunction with OEC grafts (Andrews and Stelzner, 2004; Moreno‐López et al., 2016), but have more often been used in conjunction with overexpression or knockout experiments (Andrews et al., 2009; Cafferty et al., 2004; Filous et al., 2014; Gaudet et al., 2016; Puttagunta et al., 2014), or the delivery of pharmacological agents (Qiu et al., 2005; Tedeschi et al., 2016), neurotrophins (Bradbury et al., 1999), or chondroitinase (Bradbury et al., 2002).

### Contusion Injury Models

Transection models are useful for their lesion completeness but do not very accurately reflect the majority of SCI observed in clinical settings which are more often due to impact on the vertebra as a result of car accidents, falling or sports injuries. These injuries do result in partial or complete damage to spinal cord tracts through spinal compression or displacement and without completely severing the cord. To effectively replicate the cellular response of these more common types of SCI, the contusion or transient compression model is more relevant (Fig. 2E). The contusion model relies on a defined physical impact being reproducibly delivered to the cord resulting in stretching or severing axons and injuring spinal tissue and neuronal cells depending on the force delivered.

There are multiple commercially available devices for producing contusion injuries. Among the DC lesion experiments summarized in Table 1 and Supporting Information, Table 2, two devices have been employed. The first device is the Infinite Horizon Impactor. After surgical exposure of the spinal cord and leaving the dura mater intact, a controlled impact force is delivered to the spinal cord at the desired level. When directed at the dorsal surface of the cord, the DCs can be reliably targeted with this impactor (Baker et al., 2007; James et al., 2015; Soderblom et al., 2015; Bartus et al., 2016). Another device is the MASCIS weight drop device that Pearse and colleagues used to make a moderate contusion injury by dropping a 10 g rod from a height of 12.5 mm at the mid‐thoracic level of the spinal cord (Pearse et al., 2007). The magnitude of the transient force that these devices produce on the dorsal spinal cord is decided by the investigator. This approach allows reliable comparison within experimental animals of the same study. The technical variability in the contusion paradigms used by different groups complicates comparison between studies. In a contusion lesion paradigm (Fig. 2E), it is difficult to ensure the completeness of the DC lesion, which leads to higher probability of spared fibers. Contusion models are therefore more difficult to use in studies of axon regeneration in the DC and more appropriate to use for studies of the damaged axon response in the context of a clinically relevant injury.

With respect to the DC, contusion models have been used to investigate the effect of modulating the extrinsic environment with chondroitinase (James et al., 2015), transplantation of Schwann cells (SCs), or olfactory ensheathing glial cells (OEGs; McElroy et al., 1994), in combination with a conditioning lesion (Rezajooi et al., 2004) and for the study the effect of NRG1 deficiency (Bartus et al., 2016; Supporting Information, Table 2).

## ASSESSMENT OF REGENERATION AND FUNCTIONAL RECOVERY

### Anatomy of the Ascending Sensory Afferents

The DRG contain a mixed population of sensory neurons, including large myelinated DRG neurons and small, lightly, or nonmyelinated neurons (reviewed by Lallemend and Ernfors, 2012). The large myelinated DRG neurons carry proprioceptive information from muscles and tendons and mechanoreceptive information from the skin. Upon entering the spinal cord, the axons of these neurons form a large number of collaterals that terminate in the grey matter and contact spinal interneurons and motor neurons. Some of these axons also project up the dorsal funiculi to terminate in the brainstem, forming the ascending DCs. Of these, fibers entering the cord below T6 terminate at the gracile nucleus while fibers entering above T6 terminate in the cuneate nucleus of the brain stem (Sengul and Watson, 2014). The medium to small, lightly, or nonmyelinated neurons convey mechanoreception, nociception, thermoreception, and pruriception from the skin and viscera to the dorsal horn of the spinal cord. At the higher cervical segments of the dorsal funiculus there is a larger proportion of cutaneous mechanoreceptors than deep proprioceptors, as a number of the latter leave the dorsal column at lower segments (mostly below T8) and terminate onto the dorsal nucleus, also known as Clarke's column which in turn projects to the cerebellum (Niu et al., 2013; Sengul and Watson, 2014). Approximately 25% of primary proprioceptive fibres arrive at the brainstem (Sengul and Watson, 2014).

The proprioceptive and mechanoreceptive projections from the larger, myelinated DRG neurons which travel directly to the gracile and cuneate nucleus of the brainstem do not cross the cord midline, but ascend in the ipsilateral dorsal funiculus. This is in contrast to the axons of nociceptive DRG neurons, a majority of which terminate on projection neurons in the ipsilateral dorsal horn, the projections of which then cross to the contralateral cord before ascending to the thalamus and the insular and cingulate cortices (reviewed in Basbaum et al., 2009). Despite this, ∼23% of fibers in the dorsal funiculi are unmyelinated and presumably carrying nociceptive information, and another 25% are propriospinal with the remainder presumably transmitting tactile information, discriminatory touch, vibration and conscious proprioception (Chung et al., 1987, Sengul and Watson, 2014). The upper segments of the dorsal funiculi also contain axons of a visceral pain pathway originating from the pancreas and gastrointestinal tract and potentially other viscera. These projections also terminate in the gracile and cuneate nucleus (Willis et al., 1999).

### Functional Deficits and Testing Following the Dorsal Column Lesion

The ascending sensory fibers in the DC are thought to carry the sensory modalities of tactile information, discriminatory touch, vibration, and proprioception and tests for DC function should ideally address one or more of these sensory modalities. The functional deficits that occur after DC lesion tend to be mild, and often recover spontaneously, so testing for improvement in function experimentally can be difficult, and can only be done within a short window.

Most lesion techniques also damage the CST in the dorsal funiculus, and some of the measurable deficit may be partly due to loss of CST function. Larger lesions, such as dorsal hemisection, which lesion the dorsal lateral funiculi, of course produce larger deficits. Where other tracts are damaged, if functional improvement is seen following treatment it may still be possible to ascribe this to ascending DC axons if treatment was specifically delivered to DRG neurons.

Here we summarize the functional deficits that have been established after DC lesion, in the rat unless otherwise stated.

The horizontal ladder reveals deficits after cervical DC lesion, lasting up to 6 weeks (Bradbury et al., 2002; Lu et al., 2004; Fagoe et al., 2016). However, following a specific DC lesion at C2 that left the CST intact, only a minor deficit was found up to 4 weeks, while with CST damage as well, the effect was more robust (Kanagal and Muir, 2007). A C2 DC lesion showed only forelimb deficits (Kanagal and Muir, 2008). Deficits from thoracic DC lesions are less than those from cervical lesions in this test and are often difficult to detect (Grill et al., 1997; Fagoe et al., 2016).

The tape removal test showed deficits after C4 lesion (Bradbury et al., 2002) but only minor deficits in another study (Fagoe et al., 2016), and no effect on the similar adhesive dot removal task was found after C1 lesion (Ballermann et al., 2001). Significant deficits were found on the narrow beam (Bradbury et al., 2002; Hollis et al., 2015a) and rotarod (Hollis et al., 2015b), with cervical lesions. Skilled reaching tasks have also been assessed in cervical lesions. Here either no deficit (Ballermann et al., 2001), or a deficit that disappeared rapidly (McKenna and Whishaw, 1999; Chan et al., 2005), although deficits lasting 8 weeks were found by Kanagal and Muir (2007). Incidentally, a DC‐sparing lesion of the cervical CST produced no deficit on this task (Alstermark and Pettersson, 2014). A deficit was found on a tactile discrimination task involving reaching for food (Ballermann et al., 2000). Using footprint analysis, an increase in stride length and width was found after cervical lesions (Bradbury et al., 2002), and this was also found on the Catwalk footprint analysis device (Fagoe et al., 2016).

Comparing a number of the aforementioned tests in thoracic DC lesions of varying depths in the mouse indicated that deeper lesions resulted in detectable deficits up to the sixth week (end of experiment) of measurement in a majority of tests (Hill et al., 2009). Recently we also introduced a new functional test where simultaneously tactile sensation and proprioception are evaluated in a functional testing paradigm, we dubbed the “inclined rolling ladder,” which was able to detect deficits in cervically lesioned animals up to 6 weeks (Fagoe et al., 2016) and up to 11 weeks in unpublished data with a modified version of the ladder with more rungs.

Spontaneous recovery of some function is common in rodents after partial SCI. This is thought to be due to remodeling that takes place in the spinal cord and cortex (Bradbury and McMahon, 2006). In many cases, this is aided by the fact that the animals are able to walk, which can aid recovery in a similar way to rehabilitative training. The exact mechanisms are largely unknown, although in addition the propriospinal pathway via Clarke's column, which projects to the cerebellum, will in general remain intact, and could be strengthened after a lesion. Remodeling of sensory afferents of the DRG neurons which are not in the direct/primary DC pathway and therefore spared by DC lesion, but rather terminate in the spinal grey matter and are involved in modulating the initiation and alteration of stepping patterns produced by the CPG of the spinal cord independently of supraspinal control could also potentially account for some of the spontaneous recovery seen after a DC lesion in sensorimotor tasks (reviewed in Takakusaki, 2013, 2017).

Several other studies employ sensory tests that are primarily developed for nociceptive function and were used to investigate perceptual abnormalities in treatment groups or neuropathic pain states as a result of the lesion (Chan et al., 2005; Andrews et al., 2009; Hollis et al., 2015a). For this aim, thermal and mechanical hyperalgesia can be assessed with Hargreaves and Von Frey tests. It is arguable whether either of these tests are useful in a DC lesion as local circuitry below the lesion remains intact and unconscious reflexive limb withdrawal circuits are likely still functional. Indeed, Hill et al. (2009) did not find a deficit in paw‐withdrawal latency on the Hargraves test after dorsal laceration lesions in mice, rather the opposite, as latency was decreased. These tests are undoubtedly useful in SN or dorsal root lesions where communication with local spinal circuitry is definitely severed and, possibly, to determine the presence of treatment‐induced hyperalgesia.

As is clear from the text above, evaluating CNS regeneration after DC lesion can be challenging when using functional testing, partly because deficits are quite mild and because of the spontaneous recovery. An important alternative is to use electrophysiological techniques to investigate the recovery of the neuronal circuitry.

Tan et al. (2007) performed a conditioning lesion in conjunction with antibody neutralization of NG2 chondroitin sulfate proteoglycans (CSPGs) at the DC lesion site and electrically mapped regenerating sensory axons six months postinjury by stimulating either above or below the DC lesion and recording conduction velocities at the dorsal roots (Tan et al., 2007). The regenerated axons had reduced conduction velocity, decreased frequency‐following ability, and increasing latency to repetitive stimuli.

To determine changes in the conduction velocity over a DC contusion injury at different time points with an acute electrophysiological preparation, James et al. (2011) used a method which involved separately teasing out and stimulating multiple individual axons of the dorsal roots, followed by recording conduction velocities below and above the lesion with an electrode pressed to the cord surface. The data indicated a complete conduction block at 1–7 days, and partially restored conduction at 2–4 weeks but at 3–6 months, there was no further improvement in conduction velocities. A similar method was used to demonstrate that chondroitinase ABC (ChABC) improves conduction in the DC after contusion injury (James et al., 2015).

Toft et al. (2007) stimulated the L4/5 dorsal roots and then recorded cord dorsum potentials (CDPs) caudal and rostral to an L3/4 DC lesion site and reported significantly larger CDPs in animals which had received transplants of OECs. They also measured sensory evoked potentials (SEPs) which were generated by stimulating the L4/5 dorsal roots and recording at the contralateral sensory cortex. These were also larger in transplanted animals. The measurement of SEPs can show the presence of intact sensory pathways or alternative pathways which may have formed via remodeling. For example, Bonner et al. (2011) transplanted neural precursor cells (NPCs) into a DC lesion and stimulated the sciatic nerve, measuring electrophysiological activity at the ipsilateral gracile nucleus with a 16 channel implanted electrode, they reported that the signal was relayed via the graft to the gracile nucleus with a temporal delay indicating pathway remodeling.

### Possible Pathways for Transmitting Ascending Sensory Feedback after Dorsal Column Lesion

The CL paradigm, while useful for demonstrating the potential of neuron‐intrinsic regenerative abilities, falls far short of long‐distance regeneration and functional reconnection to targets. Where functional improvement is seen after a DC lesion, it is likely therefore that interaction with and remodeling of local circuitry plays a significant role.

There is a substantial body of literature, primarily focused on the CST which describes the role of the propriospinal interneurons in recovery from spinal cord injury, reviewed by Flynn et al. (2011). Of particular interest is the fact that severed descending supraspinal connections develop new connections through de novo sprouting via propriospinal neurons which in turn re‐establish motor control over circuitry previously severed by the lesion (Bareyre et al., 2004; Courtine et al., 2008; van den Brand et al., 2012). A similar pathway of functional recovery was recently described in relation to the direct DC pathway, where a lesion at C1 of the DC was performed in conjunction with a CL paradigm. This resulted in functional recovery. Tracing revealed that the recovery was due to axotomized DC axons forming de novo connections via sprouting to spinal neurons caudal to the lesion which had intact projections to the ventral posterolateral nucleus of the thalamus (Hollis et al., 2015b). Another potential target for remodeling is the existing pathway whereby the collaterals of the ascending fibers contact Clarke's column. Local sprouting here may allow some rerouting of proprioceptive information, although this has not yet been demonstrated.

### Histological Quantification of Axon Regeneration

Axonal tracing is an essential tool for determining the degree of regeneration in spinal cord injury models. In the DC lesion model this is particularly informative because, as mentioned earlier, one can label a specific subpopulation of axons, for example, via the sciatic nerve, and the anatomy allows the identification of spared fibers by the presence of terminal labelling in the brainstem. Transganglionic labeling of DC fibers has been performed using horseradish peroxidase (HRP; Richardson and Issa, 1984), cholera toxin B‐subunit conjugated with HRP (CT‐HRP or B‐HRP; LaMotte et al., 1991; Neumann and Woolf, 1999), biotinylated dextran amine (BDA; Toft et al., 2007), Texas Red‐conjugated Dextran (DexTR; Parikh et al., 2011), microruby (Puttagunta et al., 2014), and cholera toxin B subunit (CTB; Oudega et al., 1994; Bradbury et al., 1999; Neumann and Woolf, 1999; Pasterkamp et al., 2001; Neumann et al., 2002; Qiu et al., 2002). CTB and fluorescent dextrans are currently the most commonly used tracers. Sectioning of the brainstem allows for staining of the gracile and cuneate nuclei, the point of termination of the long projecting DC afferents (Fig. 3), for axons which were spared by an incomplete DC lesion, in which case the animal can be excluded from further analysis. The histological assessment of axonal retraction or outgrowth around the lesion is often accompanied with GFAP staining to determine the borders of the lesion site, which is essential to provide a quantitative perspective on elongation of regenerative axons.

Alternatively, the expression of fluorescent proteins in DRG neurons allows their axons to be visualized without additional immunohistological procedures. This can be a substitute for transganglionic tracing if the fluorescent protein is restricted to targeted DRG. We typically inject AAVs expressing fluorescent proteins into the left L4 and L5 DRG, so that only these afferents are labelled, giving a similar effect to the delivery of tracer via the sciatic nerve. For this we use farnesylated eGFP (eGFPf), a modified, membrane‐bound form of eGFP that is axonally transported and thus efficiently labels intact, injured and regenerating fibers (Fagoe et al., 2014). Besides direct injection of the DRG, AAV vectors can be delivered intrathecally, although this will transduce multiple DRG along the cord on both sides and so does not fully substitute for transganglionic tracing (Towne et al., 2009; Fagoe et al., 2015b).

Fluorescent proteins can also be expressed using transgenic mouse lines, such as the Thy1‐GFP‐M (Tedeschi et al., 2016), ‐YFP (He et al., 2016), or ‐GCaMP (Tang et al., 2015) lines, providing fluorescent labeling of DRG neuron without the need for surgical intervention, although expression is not limited to these neurons and not specific for particular DRG. This approach has been used, for example, for two‐photon time‐lapse imaging to monitor changes in axonal networks in living mice (Dray et al., 2009; Farrar et al., 2012; Lorenzana et al., 2015; Tang et al., 2015).

The recent rise of tissue clearing techniques such as CLARITY, 3DISCO, and CUBIC, combined with fluorescent proteins and tracers promises to accelerate the histological assessment of spinal cord injury models. 3D imaging of cleared spinal cords using 3DISCO clearing and light‐sheet laser‐scanning ultramicroscopy showed the potential for this combination in the DC injury model (Ertürk et al., 2012), and this was further explored in this and other SCI models (Soderblom et al., 2015). It is expected that these techniques will become the norm in many SCI models as they allow for much more rapid anatomical assessment of sprouting and regeneration.

## EXPERIMENTAL FINDINGS OF REGENERATION IN THE DORSAL COLUMN LESION MODEL

In this section, we will first review the conditioning lesion effect as a paradigm for understanding the intrinsic regenerative response of DRG neurons. Then we will focus on the attempts to manipulate this intrinsic response to promote the regeneration of the central branch of DRG neurons following a lesion of the DC. Finally, the attempts to manipulate the lesion environment of a DC lesion, either alone or in combination with an intrinsic intervention will be discussed briefly.

### The Conditioning Lesion Effect

The CL effect, as applied in DC injury models, provides evidence that a sufficiently strong neuron‐intrinsic response can overcome some, if not all, of the inhibitory elements of the injured CNS. Here we provide an overview of the initial discovery of the CL effect and subsequent experimental attempts to decipher the underlying molecular mechanisms which result in improved axonal regeneration.

In his review on the “axon reaction,” Lieberman (1971) described the perikaryal and axonal response to axotomy, including chromatolysis and Wallerian degeneration. He noted the absence of a cell body response in DRG neurons when their central process is damaged. The lack of a cell body response following a central lesion contrasts with the extensive perikaryal reaction observed after peripheral nerve damage. The first observation of the CL effect came when it was demonstrated that the outgrowth of axons in a regenerating peripheral nerve could be accelerated by a prior “conditioning” lesion made two weeks earlier (McQuarrie and Grafstein, 1973; McQuarrie et al., 1977; Forman et al., 1980). Richardson and Issa (1984) combined a PNS CL with a subsequent lesion of the central branch of the DRG and a peripheral nerve graft. Animals which had a conditioning lesion were 100 times more likely to regenerate their transected central axons into the nerve graft, showing that injury of the peripheral axon branch promotes regeneration of the central branch. Richardson and Verge (1986) showed that the more distal the peripheral transection was from the DRG the less successful regeneration was and concluded that a possible mechanism for the CL was that it was limiting retrograde signals from the SCs, which presumably inhibited the cell body response to axotomy.

#### Factors Affecting the Conditioning Lesion Effect

To determine whether the increased intrinsic growth capacity could promote regeneration of the injured spinal cord, Neumann and Woolf (1999) performed CLs on the peripheral branch of the DRG two weeks prior, one week prior, and at the same time as a DC lesion. This article showed that a preconditioning lesion 1 or 2 weeks before a crush lesion of the spinal cord promoted growth into and beyond the lesion, the strongest effect being one week prior to injury. A CL at the time of the lesion improved sprouting into but not beyond the lesion. Since this study was published, the majority of similar studies apply preconditioning lesions one week prior to DC lesion for optimum regeneration, either as a positive control or in conjunction with another treatment (Pasterkamp et al., 2001; Cafferty et al., 2004; Han et al., 2004; Qiu et al., 2005; Cao et al., 2006; Tan et al., 2006; Alto et al., 2009; Kadoya et al., 2009; Blesch et al., 2012; Wang et al., 2012; Hannila et al., 2013; Mar et al., 2014; Puttagunta et al., 2014; Hollis et al., 2015b; Siddiq and Hannila, 2015). It is understood that a delay is necessary to allow the full expression of the RAG program which is triggered by CL, and if performed too long before a central lesion then the RAG expression may have begun to decline, although there is little data about how quickly this occurs. GAP43 mRNA returns to baseline by day 37 after sciatic nerve crush, and GAP‐43 protein in the dorsal column itself declined to baseline by 8 weeks if peripheral regeneration was permitted (Van der zee et al., 1989; Schreyer and Skene, 1991). The failure of regeneration through a lesion when the CL is performed at the same time or after the DC lesion suggests that the formation of glial scarring results in an environment that is already too prohibitive for regeneration driven by the RAG program to overcome. In support of this, laser axotomy of individual DC axons, which avoids creating an inhibitory scar, followed by a delayed CL, did result in increased regeneration (Ylera et al., 2009). Interestingly, regeneration after laser axotomy of DC axons was seen without a CL, when both central ascending and descending branches were cut (Lorenzana et al., 2015).

The CL effect in the DC has been studied further. A CL at the time of the DC lesion, followed one week later with another priming lesion results in dramatic regeneration within and beyond the lesion (Neumann et al., 2005). This suggests that a sustained regenerative state can overcome even the inhibitory nature of peri‐lesional scarring. A post CNS‐injury CL did not result in growth through the existing lesion, but it did activate the RAG program and promoted regeneration through a fresh CNS lesion, proximal to the first. This indicates that a prior central lesion does not impair the ability of sensory neurons to activate regenerative processes (Ylera et al., 2009). Peripheral nerve CL as a means to boost intrinsic regenerative mechanisms has also been used in conjunction with efforts to render DC lesions more permissive or manipulate axonal responses to the environment, often applied after or at the same time as the DC lesion.

It has been found, somewhat unexpectedly, that an L5 preconditioning ventral root transection induced sprouting of sensory fibers after a DC transection. The ventral root lesion leaves the axons of the DRG neurons intact, but caused increased brain‐derived neurotrophic factor (BDNF) expression in the DRG. The effect was not compared to a conditioning peripheral nerve lesion but appeared to be limited to sprouting at the proximal lesion border (Li et al., 2009).

Last, the genetic background was found to influence the size of the conditioning lesion effect. The CAST/Ei strain of inbred mouse was identified as having high intrinsic neuronal growth ability (Omura et al., 2015). In these mice, a CL induced considerably growth beyond a DC lesion compared to C57Bl6 mice.

#### The Properties and Behavior of Regenerating Axons after a Conditioning Lesion

Analysis of the content of central branches of the DRG following CL indicated a global increase in axon transport including an increase in anterograde transport of cytoskeleton components, metabolic enzymes and axonal regeneration enhancers (Mar et al., 2014). At longer postlesion time points, the regenerating sensory afferents of the dorsal column induced by a CL remain in a chronic pathological state as evidenced by demyelination and reduced conduction velocities (Tan et al., 2007). The regeneration of DRG axons in the DC following CL appears to be impeded by Sema3A expressing fibroblasts in the lesion scar and CNS myelin, but regenerating axons were observed growing through areas of strong Tenascin‐C and CSPG expression suggesting that CL‐induced regeneration can overcome these inhibitory molecules (Pasterkamp et al., 2001). As discussed in the section “Possible Pathways for Transmitting Ascending Sensory Feedback after Dorsal Column Lesion,” functional recovery observed after CL and a C1 DC lesion was not attributed to the elongation of the most proximal ends of the transected proprioceptive axons through or around the lesion, but rather to the formation of collateral sprouts which were terminating on spinal neurons which retained intact projections to the ventral posterolateral nucleus of the thalamus (Hollis et al., 2015b). This indicates that the CL not only induces regeneration at the cut ends of sensory axons but also induces the formation of collaterals from proximal portions of the injured axons.

#### Regeneration‐Associated Gene Expression Program

Peripheral nerve injury results in the induction of a neuron‐intrinsic gene expression program in DRG, referred to as the RAG program (see Introduction). The RAG program supports axon regeneration. It is well established that in contrast, a lesion to the central branch of DRG neurons in the dorsal root fails to induce a robust RAG program (Stam et al., 2007, Zou et al., 2009). Although gene expression changes are seen, only some RAGs are induced and with smaller magnitude (Geeven et al., 2011). Kadoya et al. (2009) showed that, as might be expected, a C3 DC lesion without CL failed to induce any significant gene expression changes in DRG neurons at 1 week or 7 weeks after injury.

### Experimental Intervention Strategies to Promote Axon Regeneration and Plasticity Following a Dorsal Column Lesion

#### Direct Manipulation of Neuron‐Intrinsic Gene Expression

A handful of studies have reported significant axonal outgrowth following direct manipulation of neuron intrinsic gene expression in DRG neurons. Transgenic mouse lines overexpressing the prototypical RAG GAP‐43 and the growth cone protein CAP‐23 under the control of the neuron‐specific Thy‐1 promoter in mice with overexpression of either gene alone DRG neurons failed to regenerate their spinal axons into a peripheral nerve graft (Bomze et al., 2001). Co‐expression of both genes triggered a 60‐fold increase in the number of DRG neurons that regenerated their spinal axons into the graft. This shows that sustained expression of two prominent growth cone components (GAP43 and CAP23) can enhance the growth competence of injured axons and can to some extent mimic the effect of a peripheral conditioning lesion.

p75NTR is a neurotrophin receptor and a co‐receptor for NOGO, a major inhibitory myelin component. Song et al. (2004) attempted to promote axonal regeneration following DC lesion by using p75NTR deficient mice, but p75NTR deficiency in the ascending DC axons was not effective in overcoming myelin associated inhibitory factors following DC lesion in vivo.

Neuronal expression of the serine protease tissue‐type plasminogen activator (tPA) has been shown to enhance axon growth both in vitro and following PNS injury. A transgenic mouse expressing tPA in conjunction with a dorsal spinal hemisection failed to promote axonal regeneration of ascending afferents or locomotor recovery following dorsal hemisection. The authors speculated that aside from the possibility that the transgenic tPA was not secreted at high enough levels, that more sensitive testing such as electrophysiology may be required to detect plasticity and remodeling changes that may not in themselves result in functional recovery after 4 weeks (Moon et al., 2006).

Overexpression of α9 integrin in DRG neurons, using directly injected AAV vector, resulted in increased penetration of DC axons into a DC crush lesion (Andrews et al., 2009). This integrin interacts with tenascin‐C, which is present in the extracellular matrix of the CNS.

The microRNA (miR)‐155–5p (miR‐155) is a noncoding RNA which negatively effects mRNA translation and had been shown to possibly alter genes that regulate axon growth in neurons and reduces macrophage associated neuroinflammation. To determine the effect of this miRNA on axon regeneration, Gaudet et al. (2016) observed enhanced axonal regeneration following transection and contusion in a miR‐155 knock‐out (KO) mouse with accompanying CL as well as reduced neuroinflammation. They also reported augmented expression of the RAG SPRR1a following a CL compared to WT neurons in miR‐155 knock‐out mice and improved locomotor function after a contusion injury. Together these data raise the possibility that miR‐155 suppresses important aspects of the neuron‐intrinsic injury response.

Metallothionein (MTI/II) is upregulated after CL and was found to allow cultured DRGs to overcome myelin associated glycoprotein (MAG) inhibition of growth. MTI/II was found to be important for the CL effect, as regeneration in the DC did not occur in MT I/II‐deficient mice after CL (Siddiq and Hannila, 2015).

#### Direct Manipulation of Neuron‐Intrinsic Gene Expression: Regeneration‐Associated Transcription Factors

In an effort to regulate a larger proportion of the RAG program and therefore promote regeneration, a number of laboratories have embraced the hypothesis that this might be achieved by direct manipulation of transcription factors (TFs). Although the full mechanism of transcriptional regulation of the RAG program is not understood, several TFs with influence on regeneration and RAG expression have been identified, mainly in peripheral nerve injury models. As the surprising discovery that fibroblasts could be reprogrammed into a pluripotent cell type, the induced pluripotent stem cell (iPSC) with the four transcription factors Oct3/4, Sox2, c‐Myc, and Klf4 (Takahashi and Yamanaka, 2006), the use of TFs to change cell phenotype has become widespread. As the induction of the regenerative phenotype is a comparable change in cell state, we and others have proposed that an appropriate combination of TFs could “reprogram” neurons into a regenerative state (MacGillavry et al., 2011; for review, see Tedeschi, 2011). Here we will summarize the findings of TF manipulation in the DC lesion.

The first TF manipulated in the DRG in conjunction with a DC lesion was the cyclic AMP (cAMP) response element binding protein (CREB) (Gao et al., 2004). CREB mediates transcriptional responses to cAMP analogs (which can partially mimic a CL; see the section “Signaling Molecules Targeting Transcriptional Pathways”) and so it was thought that a constitutively active CREB might mimic the pro‐regenerative effects of cAMP. A VP16‐CREB fusion protein was delivered to the L4 DRG in an adenoviral vector, by direct injection of the ganglion, 4 days prior to DC lesion. This resulted in a number of axons penetrating the lesion, roughly as far as the lesion center, instead of retraction from the lesion in the GFP‐only control group (Gao et al., 2004). VP16‐CREB thus induced some sprouting but the effect appears small compared to a CL.

A CL was found to cause temporally sensitive phosphorylation and activation of the TF signal transducer and activator of transcription 3 (STAT3), and when this activation was blocked in vivo, the regeneration observed after CL in the DC lesion was significantly attenuated (Qiu et al., 2005). The importance of STAT3 was explored further in an experiment utilizing in vivo imaging of severed axons in the DC of a live animal. Overexpression of STAT3 or its constitutively active variant STAT3C promoted sprouting of the central axon branches after DRG central branch injury, but only in the early stages (2–4 days after lesion) and was not able to induce sustained growth (Bareyre et al., 2011).

Based on the observation that activating transcription factor 3 (ATF3) is upregulated in all DRG neurons following peripheral branch lesion but not central branch lesion in the spinal cord, it is a reasonable assumption that ATF3 is involved in the RAG response. Transgenic animals overexpressing ATF3 in the DRG had increased speed of regeneration after peripheral nerve injury and in vitro, but ATF3 overexpression alone failed to overcome the inhibitory environment of a DC lesion (Seijffers et al., 2007).

A positive effect of ATF3 on regeneration of the central DRG axon branch was found in our laboratory in a dorsal root injury model, but regeneration only occurred until the DREZ (Fagoe et al., 2015a). We also explored whether expressing combinations of regeneration‐associated TFs might be more effective than single TFs, as TFs are known to act co‐operatively and, as mentioned above, the parallel with cellular reprogramming strategies suggests this will be necessary. We therefore combined the four RAG TFs ATF3, Smad1, STAT3, and c‐Jun. The combination also promoted faster regeneration in the dorsal root, but was not superior to ATF3 alone. Neither ATF3 alone nor the combination promoted regeneration in the DC lesion model (Fagoe et al., 2015a).

#### Signaling Molecules Targeting Transcriptional Pathways

As well as directly targeting TFs, attempts have been made to increase the growth state of DRG neurons by targeting intracellular signaling molecules that target transcriptional pathways. One of these is the second messenger cAMP which plays a central role in several downstream intracellular effector pathways (reviewed in Batty et al., 2017). Application of the membrane permeant cAMP analogue dibutyryl cyclic AMP (db‐cAMP) has been shown to reduce myelin inhibition of growth in several neuronal types (reviewed in Hannila and Filbin, 2008)), including DRG neurons (Cai et al., 2001; Qiu et al., 2002). It also leads to upregulation of some RAGs, such as Arginase I and IL6 (Hannila and Filbin, 2008). This occurs via a number of pathways, including PKA acting on CREB, PKA independent of CREB and AP1 (Ma et al., 2014). A peripheral nerve lesion doubles the levels of cAMP in DRG neurons, although after a week levels return to baseline (Qiu et al., 2002), so it is thought cAMP may help initiate the regenerative response.

The injection of db‐cAMP resulted in increased axonal growth into a DC lesion compared to controls (Neumann et al., 2002; Qiu et al., 2002). When cAMP was administered to the DRG in combination with administration of the neurotrophin NT‐3 to the lesion axons grew beyond the lesion site. This was not the case with cAMP or NT‐3 alone (Lu et al., 2004). This study is one of the first examples showing that a combinatorial strategy augmenting the intrinsic growth state and neurotrophic stimulation at the injury site promotes regeneration.

Han et al. (2004) looked more closely at the degree to which cAMP signaling mimics a CL, and found that while the injection of db‐cAMP into the DRG increased the expression of growth‐associated tubulin types as in a CL, it did not increase the velocity at which these cytoskeletal proteins are transported in injured axons as in a CL. Additionally, the injection of db‐cAMP failed to increase intrinsic growth capacity enough for axons to grow long distances through a permissive graft in the DC lesion or in a peripheral lesion. This is consistent with previous results that db‐cAMP had no effect on the rate of peripheral axonal outgrowth measured by the pinch test (McQuarrie et al., 1977).

Secretory leukocyte protease inhibitor (SLPI) was identified in a microarray screen as being strongly upregulated in response to db‐cAMP application in postnatal DRGs (Siddiq and Hannila, 2015). SLPI reduced growth inhibition by MAG in vitro, and appears to act as a transcriptional repressor in the neuronal nucleus following internalization. In the DC lesion model, CL‐induced sprouting was much reduced in SLPI null mutant mice implicating SLPI as an important component of the intrinsic CL response (Hannila et al., 2013).

The BMP‐Smad1 pathway has also been targeted in DRG neurons (Parikh et al., 2011). Smad1 expression is high in embryonic DRG neurons and declines in the adult, but is reinduced upon peripheral nerve injury. Bone morphogenetic protein (BMP) signaling through Smad1 was important for axon growth and the CL effect on neurite outgrowth in vitro. In the DC lesion model, an AAV expressing BMP4 was delivered intrathecally to target the lumbar DRG. This induced sprouting of the DC axons into the lesion. Similar effects were seen when the vector was delivered 2 weeks before or 15 min after the spinal cord lesion (Parikh et al., 2011).

Several pro‐regenerative cytokines, including LIF, IL‐6, and CNTF, signal through the gp130 receptor, which in turn leads via JAK kinases to phosphorylation of STAT3 (reviewed in Zigmond, 2012). The role of IL6 in the CL effect was investigated in IL6 knockout mice, where a CL failed to promote regeneration of the central axon projections (Cafferty et al., 2004). This was later contradicted by Cao et al. (2006) who reported that IL‐6‐deficient mice respond to a CL as well as WT mice. Furthermore, IL‐6 delivered intrathecally for 2 weeks promoted axonal growth into a DC lesion site without a CL.

#### Epigenetic Mechanisms

Alongside regulation by TFs, there is increasing evidence for a role for epigenetic changes in the RAG response. Epigenetic markers such as histone acetylation or methylation, and DNA methylation are involved in long‐term suppression of gene expression, for example, as part of cell‐type differentiation. It is plausible that genes essential to regeneration that are not normally needed in the mature neuron are packaged away tightly in heterochromatin, and that part of the CL response involves epigenetic modification of this chromatin that allows for transcriptional binding and subsequent translation of these genes.

Histone acetylation results in nucleosome disassembly and opening of chromatin and is thus pro‐transcription, and is effected by histone acetyltransferases (HATs), while histone deacetylases (HDACs) have the opposite effect. With the understanding that SMAD1, a pro‐regenerative TF, interacts with histone modifying enzymes, Finelli et al. (2013) employed two pharmaceutical HDAC inhibitors, MS‐275 and TSA which result in histone H4 hyperacetylation. This leads to the induction of a subset of RAGs in the DRG and increased axonal regeneration into a DC lesion. Puttagunta et al. (2014) screened a number of histone modifications in DRG neurons after a CL and found that acetylation of histone 3 lysine 9 (H3K9ac) occurred in selected genes after peripheral injury but not central (dorsal column) injury. The histone acetyl transferase PCAF targets this modification, and was found to be activated by CL. In the DC lesion model, CL‐induced growth into and beyond the lesion was reduced in PCAF knockout mice, while overexpression of PCAF with AAV1 injected in the sciatic nerve promoted CL‐like sprouting.

Demethylation of 5‐methyl cytosine in promoter or enhancer regions may also play a role after peripheral nerve lesion, as Tet methylcytosine dioxygenase 3 (Tet3) was found to be upregulated after a peripheral nerve lesion (Loh et al., 2017; Weng et al., 2017). This enzyme performs the first step in a methylation removal mechanism converting 5‐methylcytosine to 5‐hydroxy methylcytosine. The evidence for such changes being important for RAG regulation is mixed however (Puttagunta et al., 2014; Loh et al., 2017; Weng et al., 2017). Loh et al (2017) also studied RAG promoter methylation changes in DRG neurons in the DC lesion model, and found that despite a lack of upregulation of Tet3, such changes do occur after central lesion but at a different set of sites than after peripheral lesion, and suggest this may indicate induction of a growth‐repressed state in the injured neurons in the former case.

#### Other Methods of Inducing a Conditioning Lesion‐Like Effect

Electrical stimulation (ES) of injured nerves promotes peripheral nerve repair by and is applied in a clinical setting (Willand et al., 2016). ES, applied to the sciatic nerve, was compared to a sciatic nerve injury in a thoracic DC lesion (Udina et al., 2007). ES promoted growth into the lesion which was similar to a CL but unlike CL, failed to promote further axon elongation. Interestingly DRGs exposed to ES exhibited elevated levels of cAMP, which may partially explain the positive effects of ES. A CL‐like effect was also induced by the injection of ethidium bromide, which acts as a demyelinating agent, into the sciatic nerve. This induced c‐Jun, ATF3, and several other RAGs and resulted in greater growth into a cell graft than CL (Hollis et al., 2015b).

Direct injection of ATP into the sciatic nerve also produced a conditioning‐lesion like effect on growth into in a DC lesion, suggesting that damage‐induced ATP release might be one of the signals inducing a regenerative response in injured neurons (Wu et al., 2018). Saline injections had no effect. Two injections of ATP 1 week apart were even more effective. ATP injection also induced GAP‐43 and phospho‐STAT3 expression, but not ATF3 or c‐Jun.

### Extrinsic Factors—Acting on Injured Axons

#### Neurotrophins

Neurotrophins are secreted growth factors, often derived from target cells, which promote neuronal survival, and can also promote axon extension (neurotropism). Nerve growth factor (NGF) and neurotrophin 3 (NT3) are critical for the survival and maintenance of sensory neurons. The potential of NGF to promote axon regeneration was investigated following a DC lesion. Animals with a CL followed by DC lesion received implants of autologous denervated PNS grafts into the lesion site, and an infusion of NGF rostral to the implant. This encouraged regenerating DC axons to leave the graft and travel towards the NGF infusion site, although many axons appeared trapped there. A majority of the regenerating axons in the vehicle only group remained in the graft (Oudega and Hagg, 1996).

NT3 has been shown in the DC lesion model to encourage regeneration of sensory afferents through and beyond lesions and lesions filled with cellular grafts (Bradbury et al., 1999; Taylor et al., 2006; Hou et al., 2012). NT3 was also delivered using a regulatable lentiviral vector to the spinal cord (Hou et al., 2012). Switching off NT3 expression after 4 weeks resulted in a loss of regenerated DC axons beyond the lesion suggesting that continuous delivery is required to maintain the presence of regenerated axons beyond the lesion site.

NT3 injected directly into the nucleus gracilis in conjunction with a high cervical lesion, CL and cellular grafting of the lesion was sufficient to enable regenerating sensory axons to reach the nucleus gracilis. Electrophysiological recordings, however, did not indicate that the treatment resulted in detectable post synaptic potentials in the nucleus gracilis and functional studies were not performed. It was postulated the CTB labeled fibers beyond the lesion were unmyelinated or poorly myelinated (Alto et al., 2009).

A combination therapy targeting both intrinsic capability with bilateral conditioning lesions, the lesion environment with NT‐3 expressing mesenchymal stem cells (MSCs), and an LV‐NT‐3 gradient established rostral to the lesion resulted in bridging of the entire lesion at 6 weeks and as long as 15 months after the lesion (Kadoya et al., 2009).

Low doses of GDNF were found to enhance the conditioning lesion effect, doubling the distance grown beyond the lesion epicenter. The effect was selective for preconditioned axons, and higher doses of GDNF were not effective (Mills et al., 2007).

#### Microtubules

Generally, the regeneration‐capable severed axon endings in the PNS form spiked “growth cones” while the regeneration‐incompetent severed axon endings in the CNS form bulbous “retraction bulbs,” this bulbous morphology was found to be predominantly caused by an accretion of disorganized microtubules and the mitochondrial and *trans*‐Golgi‐network‐derived vesicles attached to them (Ertürk et al., 2007). The application of the microtubule stabilizing drug taxol resulted in less retraction bulb formation over 6 h following the lesion when directly observed in live animals using in vivo confocal imaging. Another microtubule‐stabilizing drug, epothilone B (epoB) had the additional effect of reducing the fibrotic scar and increased regeneration of ascending afferents through the scar and improved motor function (Ruschel et al., 2015). The stabilization of microtubules, combined with administration of the autophagy‐inducing peptide, Tat‐beclin1, was shown to attenuate DC axon retraction, and improved regeneration of descending CST axons (He et al., 2016). Overall, it appears that stabilization of the disturbed microtubule network following axotomy may inhibit retraction, and improve regeneration of axotomized sensory afferents.

#### Other

The intracellular signaling pathways triggered by growth‐inhibitory molecules, such as myelin components and CSPGs are thought to converge on growth‐cone Rho GTPase and its downstream effectors including Rho‐kinase (ROCK). The ROCK inhibitor Y27632 used at relatively high doses stimulated regeneration of DC axons through the lesion and at 6 weeks resulted in improvement in a number of sensory tests (Chan et al., 2005).

Conventional isoforms of protein kinase C (PKC) are also involved in the Rho‐ROCK pathway. Infusion of a PKC inhibitor after a DC lesion was reported to lead to DC axons regenerating into and beyond the lesion site (Sivasankaran et al., 2004).

### Extrinsic Factors—Modifying the CNS Environment

Some of the earliest attempts to improve the permissiveness of the lesion extracellular environment involved injecting SCs, cells known to play a significant role in supporting regeneration in the peripheral nerve. The injection of neonatal SCs induced sprouting of sensory axons in the lesion site (Li and Raisman, 1994). SCs injected into the DC lesion together with OECs resulted in partial functional recovery, although myelination of host axons was only observed in areas of SCs (Pearse et al., 2007).

Neural stem cells (NSCs) secrete neurotrophic factors including BDNF, NGF, and GDNF, which can improve host axonal regeneration after a DC lesion (Lu et al., 2003). NSCs have also been genetically modified to express and secrete NT3 (Lu et al., 2003). Fibroblasts have also been used to successfully secrete the neurotrophic factor GDNF when delivered to a lesion (Blesch and Tuszynski, 2003). Bone marrow stromal cells (BMSCs) are often grafted into the lesion site. These are pluripotent cells capable of neural differentiation and they produce NGF and NT‐3 (Lu et al., 2004). BMSCs have also been transduced to express transgenes including BDNF (Lu et al., 2004), or NT‐3 (Lu et al., 2007) or Wnt signaling inhibitors (Hollis and Zou, 2012). Glial restricted precursors (GRPs) alone or GRPs differentiated into mature astrocytes encouraged axonal regeneration into, but not out of the lesion when transplanted into the DC lesion site (Haas et al., 2012).

In the primary olfactory system, regeneration of primary olfactory axons into the CNS occurs naturally even in adults, supported by OECs. OECs express a myriad of growth permissive extracellular matrix molecules and growth factors (Blesch et al., 2012). Transplantation of OECs to the spinal cord following a DC lesion has been performed and evaluated by a number of labs. OECs do enhance regeneration in combination with a CL, enhance functional sensory recovery, and improve electrical conduction across the lesion site even in the absence of long distance regeneration (Andrews and Stelzner, 2004; Moreno‐Flores et al., 2006; Toft et al., 2007). Efforts have been made to determine the genes responsible for OECs supporting regeneration beyond those already known. The gene SCARB2 was shown to be important by overexpression in a DC lesion which resulted in improved penetration of axons into the lesion (Roet et al., 2013).

In an attempt to establish functional connectivity between the injured DC and the dorsal column nuclei (DCN), a mixture of NRPs and GRPs was transplanted into the DC lesion and a BDNF gradient established in the rostral spinal cord by injecting LV‐BDNF into the DCN. After 6 weeks, regenerating sensory axons had grown into the graft and axons originating from the graft extended to the DCN and formed synapses and functional electrophysiological activity (Bonner et al., 2011).

In an effort to increase neuronal survival following DC lesion neuroprotective immunophilin ligands were delivered subcutaneously in conjunction with CL, leading to significant sprouting and a large increase in spared fibers beyond the lesion, which indicated that the treatment reduced axon loss due to secondary injury processes at the lesion (Bavetta et al., 1999).

Myelin‐associated inhibitors of axon regeneration, including Nogo‐A and MAG, offer an interesting target for therapy following DC lesion. Delivery of soluble Nogo receptor 1 (sNgR1) fused to NGF using a lentiviral vector led to increased sprouting into the DC lesion (Zhang et al., 2013). After screening neurons in vitro for genes or molecules that allowed processes to overcome the inhibitory effects of myelin, a triazine compound F05 was found to reduce the formation of retraction bulbs and potentiated regeneration after DC lesion (Usher et al., 2010). Complement protein C1q was found to interact directly with MAG thereby reducing inhibitory signaling to neurons and enabling them to overcome myelin in culture. Following a dorsal column lesion and CL in C1q KO mice, no increase in regeneration into the lesion was seen, although there was an increase in axon turning in the lesion (Peterson et al., 2015).

The CSPGs are another class of molecule found at lesion sites that are also inhibitors of regeneration (see Sharma et al., 2012 for review). CSPGs can be digested by the bacterial enzyme ChABC. Intrathecal infusion of ChABC delivered concurrently with a DC lesion led to increased growth of DC axons around or into the lesion (Bradbury et al., 2002). ChABC also promoted sprouting of uninjured sensory afferents proximal to the lesion (Barritt et al., 2006). Delivery of LV vector expressing ChABC improved upper limb functional and electrophysiological recovery following a cervical dorsal contusion lesion (James et al., 2015). ChABC combined with a drug shown to increase myelinated tissue sparing (rolipram) and a drug used to deplete the presence of hematogenous peripheral macrophages (liposomal clodronate) led to an improvement in locomotor function compared to the individual application of each following DC lesion. The combination of a soluble Nogo receptor (NgR1) decoy, chABC, and CL allowed significant growth past the lesion, while NgR1 ectodomain, ChABC, and CL alone and combinations of two treatments were all similar, with some growth into the lesion (Wang et al., 2012).

The CSPG NG2 has been reported by different groups to be inhibitory (Filous et al., 2014), important for growth cone stabilization and guidance (Busch et al., 2010; Tan et al., 2006), and unlikely to be necessary for regeneration or functional recovery following DC lesion (Hossain‐Ibrahim et al., 2007), thus its precise role remains unclear.

Not all extracellular matrix molecules are inhibitory. Polysialic acid (PSA) is a growth permissive substrate temporarily upregulated by astrocytes in the glial scar. When PSA was expressed by lentiviral vectors astrocytes infiltrated the scar and regenerating sensory axons penetrated into the DC lesion (Zhang et al., 2007a, 2007b).

Lesion infiltration by activated macrophages has been implicated in the long distance retraction of sensory axons following DC lesion. The depletion of macrophage infiltration with liposomal clodronate or matrix metalloproteinases prevent retraction but do not by themselves promote regeneration (Horn et al., 2008). CL and chABC administration both promote regeneration and have also been shown to prevent macrophage infiltration of the lesion as an ancillary effect of treatment (Busch et al., 2009). Administration of minocycline at the time of SCI in conjunction with a CL, a macrophage deactivator, reportedly decreased the extent of axon regeneration compared to CL alone, suggesting a supportive role of macrophages in the context of CL induced regeneration (Kwon et al., 2013).

## FUTURE PERSPECTIVE

The DC lesion model has proven to be ideal for the study of the neuron‐intrinsic regenerative capabilities, how manipulation of these may be used to promote regeneration after spinal cord injury and the limits of this approach. It remains to be seen if a full recapitulation of the regenerative response can be artificially induced and sustained for a long period of time to allow long distance regeneration in this model.

The continued development of AAV vectors is likely to improve the efficiency and ease with which manipulation of gene expression can be achieved in the DRG neurons. New serotypes may make it easier to achieve high transduction rates, which are helpful when multiple transgenes need to be introduced in different vectors. A recent paper reports a transduction rate of 82% for DRG neurons after intravenous delivery, avoiding the need for invasive direct injection (Chan et al., 2017), although large‐scale virus productions would be required. Progress is being made towards reliably regulatable vectors which will allow temporal control of expression, and better simulation of natural gene expression patterns (Hoyng et al., 2014). The recent development of an AAV‐deliverable all‐in‐one CRISPR Cas system will undoubtedly allow more sophisticated manipulation of gene expression in this model (Friedland et al., 2015; Ran et al., 2015).

Functional testing in the DC lesion model still has limitations due to the often mild deficits and spontaneous functional recovery observed in rodents after DC lesion. *In vivo* live imaging has so far been used sparingly but promises to fill in the previously invisible periods between histological timepoints (Ylera et al., 2009; Lorenzana et al., 2015; Tang et al., 2015; He et al., 2016).

The use of clearing techniques in the spinal cord and 3D reconstruction of axonal tracts promises to reduce the time spent on histology and provide clearer views of imaged regenerating tracts. Clearing techniques combine well with the use of fluorescent proteins expressed in transgenic animals or delivered by AAV. Fluorescent dextran tracers might be preferable for transganglionic tracing to avoid lengthy immunostaining procedures on cleared tissue.

Further work to unravel the gene regulatory network controlling the RAG response will be necessary if a complete and sustained response is to be achieved. It is also clear that multiple levels of regulation are involved. Currently there is much interest in the role of epigenetics and indeed there is evidence that the RAG program is subject to epigenetic suppression in the intact state (Cho et al., 2013; Finelli et al., 2013). Noncoding miRNAs may also be involved in the regulation of the RAG TF response (Motti et al., 2017), with one being recently shown to negatively affect the intrinsic injury response (Gaudet et al., 2016).

Regeneration in the spinal cord may also be achieved without directly activating the RAG response. Overexpression of integrin isoforms that bind tenascin C and the integrin activator kindlin led to regeneration into and along the spinal cord after a dorsal root injury (Cheah et al., 2016). It remains to be seen whether this can overcome the barrier of a spinal cord lesion, and whether RAG expression is increased as a secondary effect.

Through the use of the DC lesion model, many insights have been gained into the cellular processes underlying a successful neuron intrinsic injury response, and how this might be used to promote regeneration after spinal cord injury. We hope this article and its accompanying tables will be a useful resource in planning DC lesion experiments or assessing outcomes observed within the model to date.
